# Hand Hygiene Practices and Microbial Investigation of Hand Contact Swab among Physiotherapists in an Ebola Endemic Region: Implications for Public Health

**DOI:** 10.1155/2017/5841805

**Published:** 2017-06-11

**Authors:** S. Ibeneme, V. Maduako, G. C. Ibeneme, A. Ezuma, T. U. Ettu, N. F. Onyemelukwe, D. Limaye, G. Fortwengel

**Affiliations:** ^1^Department of Medical Rehabilitation, Faculty of Health Sciences, College of Medicine, University of Nigeria, Enugu Campus, Enugu, Nigeria; ^2^Clinical Trial Consortium University of Nigeria, Nsukka, Nigeria; ^3^UNIRED Research Group, Hochschule Hannover-University of Applied Sciences and Arts, Hannover, Germany; ^4^Department of Nursing Sciences, Faculty of Health Sciences & Technology, College of Medicine, University of Nigeria, Enugu Campus, Abakaliki, Ebonyi State, Nigeria; ^5^Department of Physiotherapy, University of Nigeria Teaching Hospital, Ituku-Ozalla, Enugu, Nigeria; ^6^National Open University of Nigeria, Owerri Study Centre, Owerri, Imo State, Nigeria; ^7^Department of Medical Laboratory Sciences, Faculty of Health Sciences, College of Medicine, University of Nigeria, Enugu Campus, Enugu, Nigeria; ^8^German UNESCO Unit on Bioethics, Fakultät III-Medien, Information und Design, Hochschule Hannover-University of Applied Sciences and Arts, Hannover, Germany

## Abstract

**Background:**

Hand hygiene practices (HHP), as a critical component of infection prevention/control, were investigated among physiotherapists in an Ebola endemic region.

**Method:**

A standardized instrument was administered to 44 randomly selected physiotherapists (23 males and 21 females), from three tertiary hospitals in Enugu, Nigeria. Fifteen participants (aged 22–59 years) participated in focus group discussions (FGDs) and comprised 19 participants in a subsequent laboratory study. After treatment, the palms/fingers of physiotherapists were swabbed and cultured, then incubated aerobically overnight at 37°C, and examined for microbial growths. An antibiogram of the bacterial isolates was obtained.

**Results:**

The majority (34/77.3%) of physiotherapists were aware of the HHP protocol, yet only 15/44.1% rated self-compliance at 71–100%. FGDs identified forgetfulness/inadequate HHP materials/infrastructure as the major barriers to HHP.* Staphylococcus aureus* were the most prevalent organisms, prior to (8/53.33%) and after (4/26.67%) HPP, while* Pseudomonas* spp. were acquired thereafter.* E. coli* were the most antibiotic resistant microbes but were completely removed after HHP. Ciprofloxacin and streptomycin were the most effective antibiotics.

**Conclusion:**

Poor implementation of HPP was observed due to inadequate materials/infrastructure/poor behavioral orientation. Possibly, some HPP materials were contaminated; hence, new microbes were acquired. Since HPP removed the most antibiotic resistant microbes, it might be more effective in infection control than antibiotic medication.

## 1. Introduction

In clinical practice, the hand is a tool in the broad spectrum of therapy offered by physiotherapists, and its use in assessment and treatment predates modern-day physiotherapy patient care. Nevertheless, hand contact with patients, some of whom may have undiagnosed contagious diseases, makes physiotherapists, as well as the community they serve, highly vulnerable to hospital-acquired infectious diseases [1]. In an Ebola endemic region, preventive measures that break the cycle of hospital-acquired infections (HAIs) are of public health interest. This is important because Ebola virus is a highly contagious disease, which has killed 11,020 people in West Africa from 2014 to 2015 [2]. The recurrent Ebola epidemic emphasizes the necessity for healthcare infection prevention and control (IPC) practices to be applied appropriately/regularly to interrupt transmission of pathogens in healthcare settings to patients and healthcare professionals.

IPC practices target critical facility safety components, environmental decontamination, waste management, and hand hygiene [3]. In West Africa, strengthening and sustaining IPC in healthcare facilities, especially those designated for Ebola treatment, will be useful in preventing/curbing the cycle of the epidemic and its transmission across populations. The common practice is that the tertiary health institutions are the preferred reference healthcare facilities in this context. Equipment and supplies, including the provision of hand gloves, running clean water, reliable electricity, and infrastructure, are all essential elements of IPC, necessary to decrease disease transmission [3]. Therefore, in clinical practice, physiotherapists in Ebola endemic regions must observe stringent infection control protocols, especially hand hygiene practices. A review of the current hand hygiene practices among physiotherapists may provide the basis to continue or revisit existing hand hygiene practices in hospitals for the safety of the therapists, patients, caregivers, and populace. In the recent Ebola outbreak, (involving three cities: Lagos, Port Harcourt, and Enugu), Nigeria receded 20 laboratory-confirmed Ebola cases and one likely case and, in addition, traced 899 contacts that were followed up during the outbreak [4]. So far, the best preventive approach for Ebola virus disease (EVD) is stringent adherence to IPC practices, including hand hygiene practices, since scientific evidence has linked hand hygiene to a reduction in the prevalence of HAIs [5]. However, it has been reported that disease control and prevention practice in Nigerian hospitals needs to be improved [6, 7]. A recent study, in Southeast Nigeria, reported that HAIs were higher in public than in private hospitals, and while the knowledge of the workers concerning HAIs was adequate, their attitude to infection prevention was poor and significantly different [8]. In fact, HAIs are among the top ten leading causes of morbidity and mortality in hospitals in Nigeria [9], leading to increased hospital stay and cost [10, 11].

A retrospective study [12] at the University College Hospital, Ibadan, Western Nigeria, reported an overall prevalence of HAIs, for a 5-year period, as 2.6% (95% CI: 2.4–2.8) with 48.3% of all infections from surgical wards, 20.5% from medical wards, 15.1% from pediatric wards, and 16.1% from obstetrics and gynecology wards. The highest prevalence of HAIs was in surgical wards, which was at 4.4%, followed by the pediatric wards at 2.4%. Though the scenario in the physiotherapy clinic was not presented, the identified hospital wards are routinely visited by physiotherapists for patient care. Therefore, HAIs have increasingly become a source of great concern to physiotherapists and other healthcare workers/infection control officers, particularly with increasing resistance of organisms to antibiotics [13, 14]. The recent outbreak of Ebola makes it even more worrisome and requires a review of compliance to hand hygiene practices by physiotherapists who may be occupationally exposed to active infective agents. In fact, physical therapists in Nigeria contacted the World Confederation of Physical Therapists (WCPT) seeking common approach and guidance on how they can protect themselves from infections while continuing to provide services to patients. The WCPT reminded the physical therapists of its policy on infection prevention and control, which emphasizes hand hygiene practices, considering the growing concern among health workers about the outbreak of Ebola in West Africa (WCPT Newsletter 2014). Subsequently, the Nigeria Society of Physiotherapy came up with a position paper on strategies for containing the spread of Ebola virus diseases, which included stringent hand hygiene practices [15]. Earlier, the World Health Organization [16, 17] had recognized this need and launched the “SAVE LIVES: Clean Your Hands” program, which reinforces the “My 5 Moments of Hand Hygiene” approach as crucial elements of infection prevention control in care settings. This approach stipulates that healthcare workers should clean their hands, before touching a patient, before clean/aseptic procedures, after body fluid exposure/risk, after touching a patient, and after touching patient surroundings [18]. Compliance with these guidelines was investigated among physiotherapists in an Ebola endemic region in order to determine the level of awareness, compliance, and barriers to its implementation in physiotherapy departments, which is of public health interest.

## 2. Methods

### 2.1. Population and Study Design

A descriptive, cross-sectional survey design was used to study the awareness and compliance to the hand hygiene safety guidelines among physiotherapists in three tertiary health institutions in Enugu State, Southeast Nigeria. Enugu was selected for the study because it was among the three cities in Nigeria where EVD was reported [9]. Invariably, any of the three reference hospitals in Enugu would have been the likely point to access healthcare services for affected patients.

Recruitment for the study was conducted at the National Orthopedic Hospital, Enugu State University Teaching Hospital, Parklane, and University of Nigeria Teaching Hospital, Ituku-Ozalla, Enugu, respectively. Using the power analysis of 80% to detect a difference between means at an effect size of 0.46, with a significance level (alpha) of 0.05 (one-tailed), a sample size of 55 was mathematically determined. This was calculated using GraphPad Prism software (StatMate version 2.0). However, only 44 physiotherapists met the inclusion criteria and were selected from the hospitals using a simple random sampling technique. To ensure a proportional representative sample, the determined quota was allocated to each hospital based on the number of physiotherapists employed in each facility at the ratio of 4 : 2 : 1 and was in the following descending order: UNTH (25), NOHE (13), and ESUTH (6), respectively. The study process involved five stages: obtaining informed consent, ward infrastructure survey, administering the questionnaire, conducting verbal interviews, and laboratory study. The test instrument was the modified International Standardized Infection Control Questionnaire [19] and consisted of 2 main items for assessing the level of awareness of nosocomial infection and compliance with hand hygiene practices. The reliability of the instrument is 0.893 with a standardized item (interitem) coefficient of 0.881. Participants gave their written informed consent prior to data collection, and ethical approval from the University of Nigeria Health Research Ethics Committee was obtained (NHREC/05/01/2008B). All data collated were deidentified for analysis.

### 2.2. Data Collection

The researchers had the permission of the heads of physiotherapy departments, in each of the three hospitals, to approach and recruit consenting physiotherapists immediately after treating a client. Each respondent was listed under their corresponding facility until the desired number for each facility was achieved. Data were collected using modified International Standardized Infection Control Questionnaire (ISICQ), which was administered to the physiotherapists. The interview guide consisted of two sections: Section A consisted of demographic information of participants, while Section B was made up of information on various types of hand hygiene practices by the physiotherapists, compliance, barriers, and satisfaction with hand hygiene practices among physiotherapists. With the test instrument, information was elicited from the participants and then entered and verified by the researchers after further questioning during the verbal interview. Three inclusion criteria were applied as follows: (1) physiotherapists employed at the tertiary hospitals in Enugu Metropolis, (2) physiotherapists with no history of skin infection, and (3) physiotherapists that have >1-year job experience in the current facility.

To appraise physiotherapists' awareness of hand hygiene practices, the following question was asked: “Is there a hand hygiene protocol in the physiotherapy department that you are aware of?” To measure physiotherapists' compliance to hand hygiene protocols, respondents were asked, “If there is a hand hygiene protocol in your department, what do you estimate your compliance rate at?” To identify key barriers to hand hygiene practices, the physiotherapists were asked, “When you do not disinfect your hands (using soap or an alcohol handrub to kill microbes) when you should, what is the reason?” To assess physiotherapists' views on whether hand hygiene practices will prevent nosocomial infection, they were asked, “To what degree do you think there is a relationship between good hand hygiene practices and preventing hospital-acquired infections?” To measure physiotherapists' satisfaction with hand hygiene practices in their facility, they were asked, “Please rate your satisfaction with the hand hygiene practices (including glove practices) currently used at your hospital.” To measure physiotherapists' satisfaction with materials provided for hand hygiene practices in their hospital, participants were asked, “Please rate your satisfaction with hand hygiene materials currently used at your hospital.”

With this information, it was possible to understand the trends in awareness, compliance, and barriers to hand hygiene practices among physiotherapists in the hospitals. To provide an in-depth grasp of the issues already explored using the questionnaire, further information on awareness, compliance, barriers, and satisfaction with hand hygiene practices among physiotherapists was explored in focus group discussions (FGDs). The FGDs involved 15 physiotherapists who were selected from the 44 respondents using a purposive sampling technique. Three FGDs (one for each of the three tertiary hospitals in Enugu) were held with 4–6 physiotherapists, each lasting for 45–50 minutes. In the FGDs, physiotherapists' awareness, compliance, barriers, and satisfaction with hand hygiene practices, currently used in their hospitals, were explored in order to gain insights into the variable factors that might influence hand hygiene practices among physiotherapists. Verbatim responses from the FGDs were transcribed and categorized into different themes, including physiotherapists' awareness, and views on whether hand hygiene practice is effective in preventing hospital-acquired infections, which were considered as variables that might influence their compliance and level of satisfaction in this context.

### 2.3. Laboratory Tests

A laboratory study was also conducted on 19 (comprising 9, 8, and 2) out of the 44 physiotherapists from UNTH, NOHE, and ESUTH, respectively. Following a treatment session, the palms and skin folds in between the fingers of the selected physiotherapists were swabbed with sterile sticks dipped into 2 mL of sterile normal saline, prior to and after hand hygiene practices, respectively. The swabs were inoculated on MacConkey agar (Oxoid) plates and cultured by the pour-plate method. After overnight incubation at 37°C, the plates were examined for microbial growth and any visible colonies were counted and first read microscopically for morphological features (color, size, shape, edge, elevation, hemolysis on blood agar, whether there was lactose fermenting on MacConkey agar, and pigment production on Mannitol salt agar). When three or more colony forming units (CFU) were found on a plate, the organism was regarded as a bacterial contaminant. Further identification of organisms was done after they were subcultured on fresh nutrient agar. Bacterial isolates were identified based on colonial morphology, Gram stain, and a battery of biochemical tests including catalase, coagulase, oxidase, indole, Voges-Proskauer, and motility tests. All the laboratory methods were done as described by Collins and Lyne [20]. Subsequently, the bacterial isolates that were identified on the agar plates were counted. A bacterial count of ≥1 × 10^5^ per mL was considered as significant, while counts of less than 1 × 10^5^ per mL were considered as no significant bacterial growth [21]. The bacteria count was done manually after serial dilution of the inoculums using standard techniques described by Miles et al. [22]. Thus, CFU/mL = (number of colonies × dilution factor)/volume of culture plate. The Petri dish was subsequently set on a gridded background. The cells in each grid cell were counted, moving in a methodical pattern through all of the cells. The counted colonies were marked on the back of the Petri dish, and a minimum of three plates were counted. Only plates containing 30 to 300 colonies were counted for vigorous inferences. An antibiogram of the 18-hour pure cultures of the isolates was obtained also using the disc diffusion method carried out on Diagnostic Sensitivity Test (DST) agar as described elsewhere [23]. The Gram-negative discs contained the generally used antibiotics in Nigeria, which included cephalexin, trimethoprim, pefloxacin, nalidixic acid, gentamicin, ampicillin-cloxacillin, streptomycin, ampicillin, ofloxacin, and ciprofloxacin. The Gram-positive discs contained ciprofloxacin, norfloxacin, gentamicin, lincomycin, streptomycin, rifampicin, flucloxacillin, erythromycin, chloramphenicol, and ampicillin-cloxacillin. The antibiotic discs were tightly fixed on the DST agar plates earlier lawn-seeded with the standardized inocula. After 24-hour incubation at 37°C, zones of inhibition were measured with a ruler calibrated in millimeters.

### 2.4. Statistical Analysis

Qualitative data were analyzed thematically while quantitative data were analyzed using descriptive statistics. The frequency of usage of hand hygiene materials was categorized as almost all the time (81–100%), not as often (61–80%), sometimes (41–60%), occasionally (21–40%), and once in a while (0–20%). Physiotherapists' satisfaction was categorized as satisfied, somewhat satisfied, neutral, somewhat dissatisfied, and dissatisfied. Item-by-item analysis was carried out to show the frequency of response and percentages of various categories of data. Distribution of species of microbial isolates found on surface contact swab taken from the hands of physiotherapists, in various hospitals, was presented in percentages.

## 3. Results

Of the 44 respondents, 23 (52.3%) were males and 21 (47.7%) were females, comprising 9 (20.50%) intern physiotherapists, 25 (56.80%) senior physiotherapists, 6 (13.60%) principal physiotherapists, 2 (4.50%) assistant directors, and 2 (4.50%) deputy directors (Table 1). Most of the physiotherapists were specialized in orthopedics and sports physiotherapy.

### 3.1. Ward Infrastructure Survey

**Figure 1:**
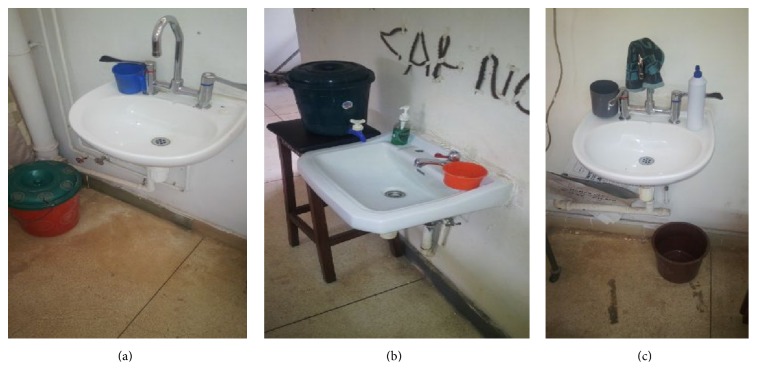
(a) National Orthopedic Hospital Enugu; (b) University of Nigeria Teaching Hospital; (c) Enugu State University Teaching Hospital.

Ward infrastructure survey (Table 2 and Figures 1(a), 1(b), and 1(c)) showed that there were inadequate infrastructures and materials required to achieve the desired level of hand hygiene in all the physiotherapy departments across the three tertiary hospitals. Such materials include sinks, clean running water, 500 mL wall-mounted dispensers for alcohol-based handrub, liquid soap, printed material, reminders in the workplace, e.g., posters, educational tools (leaflets, brochures, handouts of hand hygiene training slides, etc.), and advocacy documents for senior managers/administrators such as evaluation tools (e.g., ward infrastructure survey, hand hygiene observation). In fact, apart from the sink and bar soap, every other required infrastructure recommended by the WHO was not provided.

The majority (34/77.3%) of the physiotherapists agreed that there was a hand hygiene protocol in their department (Table 3); however, compliance with the established hand hygiene protocol by physiotherapist scored 71–100% by less than half (15/44.1%) of the respondents. Most (14/31.8%) of the physiotherapists identified “forgetfulness” as the major reason why they did not wash/disinfect their hands (using soap or an alcohol handrub) when they should. Similarly, the same number of physiotherapists (14/31.8%) also identified the distant/inconvenient location of the hand hygiene materials (i.e., sink, disinfectants, and hand wash) as the major barrier to compliance with hand hygiene practices. Furthermore, 17 (38.6%) and 16 (36.4%) physiotherapists, respectively, agreed that there is a strong and very strong relationship between good hand hygiene practices and prevention of HAIs, respectively. However, less than half of the physiotherapists indicated that when working with colleagues who forgot to disinfect their hands before touching a patient, they reminded them to do so 41–70% of the time. Overall, less than half of the physiotherapists were somewhat satisfied with the hand hygiene practices. Similarly, less than half of the physiotherapists were somewhat satisfied with hand hygiene materials provided in their hospitals.

Furthermore, half of the physiotherapists indicated that they used soap and water to wash their hands 81–100% of the time after treating a patient while none agreed that they used no hand hygiene materials 81–100% of the time (Table 4). Interestingly, almost all (43/97.7) the physiotherapists agreed that they did not use any of the hand hygiene materials 0–20% of the time (Table 4). However, the majority (36/81.8%) of them agreed that they used both (i.e., alcohol gel or foam alone and water and soap) 0–20% of the time, whereas a little more than half (26/59.1%) of them agreed that they used only soap and water 0–20% of the time.

The laboratory study showed that about 5 species of microbes were isolated from all the specimens taken from the physiotherapists prior to hand hygiene practices (Table 5). They included* Staphylococcus aureus, coagulase *+ve* Staphylococcus, E. coli, Bacillus *spp., and* Pseudomonas *spp. Overall, 15 and 11 specimens recorded significant growth of microbial colonies prior to and after hand hygiene practices in the same individuals, respectively. A breakdown of these figures across the various hospital groups showed that 9, 4, and 2 specimens from the UNTH, NOHE, and ESUTH recorded significant growth of microbial colonies prior to hand hygiene practices, respectively. Of this number, only specimens from 2 (out of 8) physiotherapists from the UNTH had no significant growth of any microbial colony after hand hygiene practices (Table 6), whereas 5, 4, and 2 specimens from the UNTH, NOHE, and ESUTH showed significant growth of microbial colonies after hand hygiene practices, respectively. However, it was observed that while some of the microbes earlier identified were removed after hand hygiene practices, new ones were acquired. For instance, among physiotherapists at the UNTH, 1 new species of microbes (*Pseudomonas *spp.) that was not found prior to hand hygiene practices was isolated from the specimens taken from the same physiotherapist after hand hygiene practices. Similarly, 2 new species (*Bacillus *spp. and* Pseudomonas *spp.) were identified from specimens collected from 2 different physiotherapists after hand hygiene practices at the NOHE.

Overall, the profile of the isolates indicated that* S. aureus* was the commonest colony while* Pseudomonas *spp.*, Bacillus *spp., and* E. coli *were the least common microorganisms isolated from the hands of the physiotherapists. Among physiotherapists in ESUTH, it was observed that the same microbial colony found prior to hand hygiene practices was isolated from the specimens obtained from the same individuals after hand hygiene practices. In contrast,* E. coli*, which was only found in a specimen from the UNTH prior to hand hygiene practices, was not present in the specimen taken from the same physiotherapist after hand hygiene practices. Furthermore, it was observed that hand hygiene practices did not change the carriage of microbes in the hands of physiotherapists at the ESUTH (Table 7), unlike in the UNTH and NOHE. In all the hospitals, apart from ESUTH, the carriage of microbes in the hands of physiotherapists decreased except in the cases where new species of microbes were acquired after hand hygiene practices.

The antibiotic sensitivity testing indicated that the bacterial isolates were resistant to most of the available antibiotics (Table 8). Isolates of* E. coli* showed the highest levels of resistance and were susceptible to only two of the antibiotics (ciprofloxacin and streptomycin).* Staphylococcus aureus* showed the least resistance, being susceptible to ciprofloxacin, norfloxacin, gentamicin, lincomycin, streptomycin, ofloxacin, and pefloxacin. Ciprofloxacin and streptomycin were the most effective antibiotics against all microbes.

### 3.2. Focus Group Discussions

There were 15 participants for the FGDs, with an age range of 22 to 59 years. Most of the participants stated that they were aware of hand hygiene protocols and guidelines required in the hospital, but sometimes they do not comply fully with the procedure for a number of reasons. Some of the reasons mentioned included lack of clean running water, soap, liquid soap dispensers, alcohol gel or foam, disposable hand towels, inconvenient location of the sinks from the treatment rooms/areas, forgetfulness, and work pressure associated with the high patient load. Most of the physiotherapists stated that their hospital did not satisfactorily provide the required materials for hand hygiene practices. One of them stated the following:

…as far as I know,…our (Physiotherapy) department has no liquid soap dispensers…no clean running water…In fact, the other day, I could not wash my hands, because there was no water in the storage tank, and alternatively, the janitor fetched some brown-coloured rainwater from a broken container kept outdoors for ages…I was afraid that there could be more contaminants in that water than I could imagine…however, some of my colleagues often washed their hands with such water

One physiotherapist stated that since he regularly wore disposable hand gloves while treating his patients, he did not see the need to wash his hands thereafter. In his view, wearing hand gloves provides sufficient barrier to break the cycle of infection transmission within the hospital. However, other physiotherapists shared contrary views. They claimed that the hand gloves often got torn due to the poor quality of the gloves that were often provided by the caregivers (since the hospital stores usually had no stock). Thus, they still saw the need for hand hygiene practices. One of the physiotherapists stated the following:

…we use whatever disposable hand gloves that were made available by caregivers (i.e. patient relatives)…Often times, these gloves were of low quality, and got torn while making vigorous contact with the patients during physiotherapy procedures;…

For some physiotherapists, the greatest barrier to hand hygiene practices was the inconvenient location of the sink and forgetfulness. Some physiotherapists claimed that they had only one sink for general use within their department, while some others claimed that extra sinks were exclusively provided in the offices of the heads of departments and a few senior practitioners. However, they all agreed that the number of sinks in their departments was inadequate and they were not located in the treatment areas or rooms. They recounted the difficulty they experienced shuttling between the treatment rooms and the location of the sink, after each treatment session. They stated that such repeated trips got more tasking when the number of patients scheduled for physiotherapy treatment was high. At some point, the physiotherapists apparently got burnout and failed to comply with hand hygiene protocols. One of the physiotherapists stated the following:

…the sink…is not located within reach from the treatment area…and that is the greatest problem I encountered whenever I needed to wash my hands after treating a patient…As a result of this situation, I found it convenient to wash my hands after treating the last patient on appointment…

Overall, a number of physiotherapists were somewhat satisfied with the level of compliance with hand hygiene practices in their department. They attributed their views to several factors including the occasional provision of soap and water and a change in the attitude of the hospital management that adopted the WHO hand hygiene protocols as a standard treatment procedure to protect staff and patients. One of the physiotherapists stated the following:

…we are somewhat satisfied that hand hygiene protocols have been adopted by our hospital…and over time, we expect remarkable improvement in its implementation

However, most of them held a contrary view, and one of them stated the following*:*

hand hygiene practices in my department is not satisfactory…because we were never provided with alcohol gel or foam…we hardly got a regular supply of soap and clean running water…With the advent of Ebola, I resorted to the personal purchase of water packaged in sachets, and alcohol gel, rather than wait for them (hospital management) to do the needful…which they rarely did

## 4. Discussions

The majority of the physiotherapists agreed that there was a defined protocol and procedure for hand hygiene practice in their various hospitals/departments. This finding agrees with the results of another study in Southeast Nigeria [8]. It suggests the possibility that physiotherapy services provided in some hospitals within the Ebola endemic regions were done while recognizing the need for hand hygiene practices and adopted certain acceptable hygienic standards. However, a “buy-in” of relevant practitioners to this policy could be a key challenge to its implementation. Apparently, a “buy-in” was achieved for the most part as the majority of the physiotherapists agreed that there is a link between hand hygiene practices and prevention of HAIs. This realization was expected to translate into a high level of compliance with hand hygiene practices by the physiotherapists. In contrast, less than half of the physiotherapists self-reported a compliance rate of 70–100% with hand hygiene practices after a treatment session. This might suggest that there were other intervening factors that did not necessarily include self-discipline or other behavioral components required for compliance with the hand hygiene protocols.

It is, however, worrisome that as many as 11 (25%) physiotherapists were not convinced of a causal relationship between hand hygiene practices and prevention of HAIs. In fact, a physiotherapist did not comply with hand hygiene practices most of the time, because he was “unsure of the need” (Table 3). This scenario could suggest a gap in knowledge on hand hygiene practices based on the level of evidence relevant for practice. Considering the adverse public health implications of poor hand hygiene practices in an Ebola endemic region, greater attention must be focused on consistent and accurate implementation of IPC practices [3] by all health facilities.

### 4.1. Strengths and Weaknesses of the Study

The present study holds strength in the fact that majority (44 out of 67) of the physiotherapists employed in the three tertiary hospitals were studied, and all cadres were represented. However, the sample size was small and could be a weakness considering the likely margin of error. The level of physiotherapists' awareness, compliance, barriers to compliance, and satisfaction with availability of hand hygiene materials/practices in their respective hospitals was simultaneously investigated in a well-described population. This might be relevant in understanding the perspectives of physiotherapists on the weakest chain in the infection transmission cycle within their locality that might hold a public health concern in an Ebola endemic region. The hospital-based design allowed simultaneous presentation of views/opinions from physiotherapists with different experiences on hand hygiene practices after patient treatment and controlling for key factors, including a tier of health facility (tertiary versus general hospital), employment duration, and history of skin infection.

However, the study may have some limitations, because the cross-sectional design suggests that it is difficult to infer causality between microbial loads on the hands of physiotherapists and bodily contact with patients. This is plausible because the microbial loads on the hands of the physiotherapists were not determined prior to patient treatment but were only assessed prior to and after hand hygiene practices. In addition, it is difficult to infer whether physiotherapists' satisfaction, with compliance to hand hygiene practices in their hospitals, was affected by past experiences when no such protocols existed rather than taking reference from the prescribed standards for IPC practices. Over time, as physiotherapists gain work experience, longitudinal observations on hand hygiene practices would be possible but were not explored in this study. Moreover, the levels of awareness, compliance of physiotherapists to hand hygiene practices, and satisfaction with compliance with hand hygiene practices in hospitals were self-reported and not objectively measured, which may limit the accuracy of measurements. Moreover, memory recall over time may not be so accurate. However, the test instrument validated self-reporting, which allows these measures to be applied to large numbers of people. Awareness and satisfaction are established influential factors on behavior, and these results might have a translational value for the health-seeking behavior of physiotherapists. In spite of these limitations, the strengths of the study suggest that it has both scientific and practical implications.

### 4.2. Relevance of Findings to the Field

Most of the time (81–100% of the time), half of the physiotherapists used soap and water in hand hygiene practices, whereas only 2 (4.55%) of them used alcohol gel or foam alone, which are disinfectants prescribed for IPC practices [3]. In fact, wall-mounted dispensers for alcohol-based handrub were not provided in any of the physiotherapy departments as indicated in the ward infrastructure survey. Similarly, wall-mounted liquid soap dispensers were also not provided. The implication is that either physiotherapists were not using hand gloves as often as they were required to or they were using it inappropriately. This is reasonable because it is recommended that when the hands are not visibly dirty, an alcohol-based handrub should be used to routinely decontaminate the hands before donning sterile gloves [24–26]. Thus, if physiotherapists adhered to hand hygiene practices in all these contexts, they would have used alcohol gel or foam alone more frequently than they claimed. However, the weakest link in the chain of infection control among physiotherapists could be the hand hygiene practices they adopted 0–20% of the time, whereby majority (43 out of 44) of them did not comply with any form of hand hygiene practices after treating their patients. It is plausible that, in such situations, forgetfulness to wash their hands and nonprovision of hand hygiene materials might be key factors as already stated by the physiotherapists in FGDs. For instance, most of the participants stated that there was no clean running water in their facilities, and they were usually provided with water from unhygienic sources, as an alternative. This view is supported by the ward infrastructure survey, which revealed the obvious inadequacy of hand hygiene materials in all the physiotherapy departments. Perhaps, it was the unavailability of soap and water that informed the observed preference of more physiotherapists (36) to use alcohol rub or foam alone 0–20% of the time, whereas they ordinarily preferred soap and water most (i.e., 81–100%) of the time. In the same context (i.e., 0–20% of the time), almost all (43 out of 44) the physiotherapists did not observe any form of hand hygiene practices, which validates the physiotherapists' claim in FGDs that when water from unhygienic sources was provided, they preferred not to wash their hands. Therefore, it is also plausible that the physiotherapists that used alcohol gel or foam alone procured them with their personal resources since the ward infrastructure survey indicated that no such materials were provided by the hospitals. Invariably, the hand hygiene practices of physiotherapists 0–20% of the time should be of grave clinical significance in an Ebola endemic region, because HAIs are more likely to be transmitted in such contexts, and must be revisited by hospital facility managers. The above scenario might explain why only less than half of the physiotherapists indicated that they were either satisfied or somewhat satisfied with hand hygiene materials currently used at their hospitals.

It was revealed that, in less than 40% of the time, the majority of the physiotherapists reminded their colleagues or were reminded by their colleagues to disinfect/decontaminate their hands when they forgot to do so after treating a patient. This is obviously inadequate and highlights the relevance of using reminders to ensure compliance with hand hygiene practices (e.g., posters and educational tools such as leaflets, brochures, and handouts of hand hygiene training slides) at the workplace, which were not available in any of the physiotherapy departments.

It was further revealed that awareness of hand hygiene practices may not necessarily translate into the desired level of compliance; otherwise, more than half of the physiotherapists, who had earlier claimed to be aware of hand hygiene practices, should have also complied with them. A similar trend has been reported in another study conducted in hospitals within the same locality as this study [8]. Invariably, in health-seeking behavior, awareness of preventive options alone may not induce appropriate behavioral responses. There could be other modifiers. For instance, the majority of the physiotherapists identified forgetfulness and unavailability of hand hygiene materials, respectively, as the commonest reasons why they failed to comply with hand hygiene practices. An erroneous conclusion might be that majority of physiotherapists did not regard hand hygiene practices as an important step towards disease prevention; otherwise, they would rarely forget. On the contrary, frustration from persistent lack of hand hygiene materials, apart from infrastructural dysfunction (such as the inconvenient location of the sink from treatment areas), might induce selective amnesia as predicted by the mnemic neglect model [27]. It further emphasizes the usefulness of reminders in ensuring consistent and accurate implementation of IPC practices in clinical settings [3], which was not found in any of the physiotherapy departments, during the ward infrastructure survey.

Laboratory study revealed that, prior to hand hygiene practices, the carriage rate of* S. aureus *(the commonest microbial isolates) was 47.37%, which agrees with a previous observation that carriage rates of* S. aureus* are 25% to 50% and might be equivalent or higher in healthcare workers than in the general population [19, 27]. The presence of* S. aureus* in hand contact surface swabs has clinical implications for patients treated by physiotherapists because its transmission occurs by direct contact with a colonized carrier [19, 21, 27, 28]. Therefore, patients with unhealed surgical wounds, when treated by physiotherapists that are colonized carriers, should be at increased risk of developing surgical site infections with* S. aureus* [29]. Moreover,* S. aureus* has been linked with urinary tract infection, which is the commonest nosocomial infection reported in another study in Southeast Nigeria [8]. This could be of clinical significance for physiotherapists that eat finger foods. This is plausible because* Staphylococcus aureus* is capable of producing several enterotoxins (SEs) with intoxication symptoms of variable intensity in humans when ingested through contaminated food [30]. Further observations that, in some cases, the hands of colonized carriers were not free of significant microbial loads after hand hygiene practices do not necessarily suggest the ineffectiveness of hand hygiene practices but might imply that the right procedures were not followed in its implementation. This was particularly observed at the NOHE, where the carriage of the microbes among physiotherapists was not changed after hand hygiene practices. This suggests the need for a comprehensive review of the implementation of the hand hygiene protocols in that hospital.

The variation in the frequency of distribution of bacteria isolated from the hands of physiotherapists across the three hospitals might be related to the methods/techniques applied in handling and treating of the patients. Similarly, the bioburden of pathogens differs along with different activities of patients such as coughing, sneezing, and loud talking while in pain/discomfort [31]. These activities eject pathogens in the environment, some of which might adhere to the hands of physiotherapists. A similar trend has been reported elsewhere [31]. Across the three hospitals, disinfecting the hands of physiotherapists after hand hygiene practices was least achieved at the ESUTH. This might imply either that the hand hygiene materials were inadequate or that the procedures adopted in these hospitals were inappropriate, or both, and must be reviewed. In fact, most of the participants in the FGDs observed that dirty rain water was provided for hand hygiene practices in some of the hospitals, which was validated by the ward infrastructure survey, which revealed that none of the three hospitals had clean running water in the physiotherapy departments. It is possible that the rain water or bar soap used in hand hygiene practices might harbor the variously identified pathogens. This might explain why new pathogens were acquired and, therefore, isolated from the hands of physiotherapists after hand hygiene practices at the UNTH and NOHE. Furthermore, a specimen from the UNTH revealed the significant growth of* E. coli *(indicative of fecal contaminants) [32], prior to hand hygiene practices.* E. coli *had also been found in stroke patients managed by physiotherapists in another study [1], which might be related to the inadequacy of the patients' personal hygiene care considering their known functional limitations [33]. However, unlike other microbial colonies, the hands of the physiotherapists were disinfected of* E. coli* after hand hygiene practices.

### 4.3. Implications for Care Teams and Policymakers

The role of hospital administration in ensuring compliance with hand hygiene practices was highlighted by the observation that the majority of the physiotherapists did not routinely disinfect their hands before treating a new patient. This scenario was compounded by the inability of the facility managers to routinely provide the required hand hygiene materials. This trend was identified by most of the physiotherapists as one of the two most important barriers to hand hygiene practices. The flaws in infrastructure design of the physiotherapy departments were highlighted by the observations that there were no sinks in the treatment areas of the three physiotherapy departments. Moreover, the provision of only one sink, at an inconvenient/distant location from the treatment area, is an aberration that was identified as a barrier to hand hygiene practices. These scenarios demand the intervention of the relevant health regulatory authorities, to correct these anomalies during facility accreditation visitation to hospitals. Overall, there could be obvious operational and logistic constraints that warranted poor provision of basic municipal services, such as clean running water, in these hospitals. These constraints must be addressed by policymakers and hospital administration to break the cycle of hospital-acquired infections, in an Ebola endemic region. Furthermore, for the sake of standardization, hospital stores department must be funded to procure acceptable quality disposable hand gloves, rather than the caregivers. This is also relevant in addressing the observation by physiotherapists in FGDs that hand gloves procured by the caregivers had no specific standards and often got torn during physiotherapy procedures.

## 5. Conclusion

Hand hygiene practices were not satisfactorily implemented in some hospitals, which explains why the identified microbes were not totally removed; rather, new ones were acquired after hand hygiene practices. The barriers to proper implementation of hand hygiene practices were identified as inadequate hand hygiene materials, poor infrastructure, and behavioral orientation. The fact that some of the microbes were resistant to antibiotics is a major concern for public health since this could lead to an increased incidence of treatment failure and severity of the disease [34]. Globally, it has been acknowledged that the treatment of infections is less successful with the emergence of bacteria that are resistant to multiple antibiotics [35]. This scenario is likely to be compounded with the outbreak of Ebola. Given that some antibiotic resistant microbes were disinfected from the hands of physiotherapists after hand hygiene practices, then hand hygiene practices might be more effective in infection control than antibiotic medication and could be of public health importance in an Ebola endemic region. Therefore, these findings highlight the need to review policy strategies required to address infrastructural gaps, continuing professional education, and attitudinal change required for effective implementation of hand hygiene practices in physiotherapy departments, within Ebola endemic regions. Otherwise, it is not difficult to imagine that an outbreak of Ebola virus disease in the present state of affairs might have a far-reaching adverse public health impact.

## Figures and Tables

**Table 1:** Sociodemographics of the respondents.

Demographic characteristics	Frequency	Percentage (%)
Gender		
(i) Male	23	52.3
(ii) Female	21	47.7

Rank of respondent		
(i) Intern	9	20.5
(ii) Senior PT	25	56.8
(iii) Principal PT	6	13.6
(iv) Assistant director	2	4.5
(v) Director	2	4.5

Unit of specialty		
(i) Medicine	3	6.8
(ii) Outpatient	2	4.5
(iii) Orthopedics	8	18.2
(iv) Other (O&G, NDT, adult neuro)	31	70.5

O&G: obstetrics and gynecology; NDT: neurodevelopmental therapy; neuro: Neurology.

**Table 2:** Hand hygiene observation and ward infrastructure survey.

Materials for hand hygiene practices in healthcare setting	ESUTH	NOHE	UNTH
Other items			
(i) Sinks	✓	✓	✓
(ii) Clean running water	0	0	0
(iii) 500 mL wall-mounted dispensers for alcohol-based handrub	0	0	0
(iv) Liquid soap	✓	0	✓
(v) Printed material	0	0	0
(vi) Reminders in the workplace (e.g., posters) Educational tools (leaflets, brochures, handouts of hand hygiene training slides, etc.)	0	0	0

Advocacy documents for senior managers			
(i) Evaluation tools (e.g., ward infrastructure survey, hand hygiene observation)	0	0	0

**Table 3:** Barriers to compliance with hand hygiene guidelines among physiotherapists.

Question (?)	Number	(%)
Is there a hand hygiene protocol in the physiotherapy department that you are aware of?		
(i) *Yes*	34	77.3
(ii) *No*	5	11.4
(iii) *Do not know*	5	11.4

If there is a protocol, what do you estimate your compliance rate at?		
(i) *Never*	1	2.3
(ii) *1–10%*	1	2.3
(iii) *11–40%*	6	13.6
(iv) *41–70%*	17	38.6
(v) *71–100%*	19	43.2

When you do not disinfect your hands (using soap or an alcohol handrub to kill microbes) when you should, what is the reason?		
(i) Too busy	6	13.6
(ii) Forget	14	31.8
(iii) Unsure of need	1	2.3
(iv) Out of product(s)	14	31.8
(v) Product(s) not in convenient location	9	20.5

To what degree do you think there is a relationship between good hand hygiene practices and preventing hospital-acquired infections?		
(i) Very weak	1	2.3
(ii) Weak	4	9.1
(iii) Neither weak nor strong	6	13.6
(iv) Strong	16	36.4
(v) Very strong	17	38.6

When working with a caregiver and you forget to disinfect your hands before touching a patient, what percent of the time does your colleague remind you?		
(i) Never	12	27.3
(ii) 1–10%	10	22.7
(iii) 11–40%	14	31.8
(iv) 41–70%	5	11.4
(v) 71–100%	3	6.8

When working with a colleague who forgets to disinfects his/her hands before touching a patient, what percent of the time do you remind them?		
(i) Never	11	25.0
(ii) 1–10%	9	20.5
(iii) 11–40%	8	18.2
(iv) 41–70%	11	25.0
(v) 71–100%	5	11.4

Please rate your satisfaction with the hand hygiene practices (including glove practices) currently used at your hospital		
(i) Dissatisfied	5	11.4
(ii) Somewhat dissatisfied	1	2.3
(iii) Neutral	5	11.4
(iv) Somewhat satisfied	17	38.6
(v) Satisfied	16	36.4

Please rate your satisfaction with hand hygiene materials currently used at your hospital		
(i) Dissatisfied	7	15.9
(ii) Somewhat dissatisfied	9	20.5
(iii) Neutral	7	15.9
(iv) Somewhat satisfied	18	40.9
(v) Satisfied	3	6.8

**Table 4:** Usage of hand hygiene materials by physiotherapists (*N* = 44).

How often (%) do you use these products to disinfect your hands after treating patients?				
% usage after treating a patient	Soap and water	Alcohol gel or foam alone	Both	Neither
0–20%	6 (13.6)	26 (59.1)	36 (81.8)	43 (97.7)
21–40%	2 (4.5)	6 (13.6)	2 (4.5)	1 (2.3)
41–60%	6 (13.6)	6 (13.6)	1 (2.3)	0 (0.0)
61–80%	8 (18.1)	4 (9.1)	2 (4.5)	0 (0.0)
81–100%	22 (50.0)	2 (4.5)	3 (6.8)	0 (0.0)

**Table 5:** Distribution of microbes isolated from physiotherapists at three tertiary hospitals (*N* = 19).

Isolates	UNTH (9 PTs)		NOHE (8 PTs)		ESUTH (2 PTs)		Total (19 PTs)	
	B	A	B	A	B	A	B	A
	*N*(%)	*N*(%)	*N*(%)	*N*(%)	*N*(%)	*N*(%)	*N*(%)	*N*(%)
*S. aureus*	4 (80.0)	1 (20.0)	3 (60.0)	2 (40.0)	1 (50.0)	1 (50.0)	8 (100.0)	4 (50.0)
*Coagulase *+ve* Staphylococcus*	3 (60.0)	2 (40.0)	1 (100.0)	0 (0.0)	1 (50.0)	1 (50.0)	5 (100.0)	3 (60.0)
*E. coli*	1 (100.0)	0 (0.0)	NSBG	NSBG	NSBG	NSBG	1 (100.0)	0 (0.0)
*Bacillus *spp.	1 (50.0)	1 (50.0)	0 (0.0)	1 (100.0)	NSBG	NSBG	1 (100.0)	2 (200.0)
*Pseudomonas *spp.	0 (0.0)	1 (100.0)	0 (0.0)	1 (100.0)	NSBG	NSBG	0(0.0)	2

*S. aureus*: *Staphylococcus aureus*; *coagulase* +ve: coagulase-positive; *E. coli*: *Escherichia coli*; spp.: species; PT: physiotherapist; B: before treatment; A: after treatment; %: frequency of occurrence; NSBG: no significant bacterial growth; NOHE: National Orthopedic Hospital Enugu; UNTH: University of Nigeria Teaching Hospital; ESUTH: Enugu State University Teaching Hospital.

**Table 6:** Total bacterial count from physiotherapists at three tertiary hospitals (*N* = 19).

Isolates	Cumulative microbial load (CFU) before treatment	Cumulative microbial load (CFU) after treatment	Cumulative difference (CFU)	Percentage (%)
*S. aureus*				
(i) UNTH	620,000	111,000	509,000	82.1
(ii) NOHE	402,000	292,000	110,000	27.4
(iii) ESUTH	189,000	151,000	38,000	20.1
*Coagulase* +ve* Staphylococcus*				
(i) UNTH	435,000	303,000	132,000	30.3
(ii) NOHE	152,000	Nil	152,000	100.0
(iii) ESUTH	121,000	119m 000	2,000	0.2
*E. coli*				
(i) UNTH	127,000	Nil	127,000	100.0
(ii) NOHE	NSBG	NSBG	NSBG	NSBG
(iii) ESUTH	NSBG	NSBG	NSBG	NSBG
*Bacillus *spp.				
(i) UNTH	123,000	117,00	6,000	4.9
(ii) NOHE	Nil	127,000	−127,000	Nil
(iii) ESUTH	NSBG	NSBG	NSBG	NSBG
*Pseudomonas* spp.				
(i) UNTH	Nil	153,000	−153,000	Nil
(ii) NOHE	Nil	126,000	−126,000	Nil
(iii) ESUTH	NSBG	NSBG	NSBG	Nil

*S. aureus*: *Staphylococcus aureus*; *coagulase*  +ve: coagulase-positive; *E. coli*: *Escherichia coli*; spp.: species; PT: physiotherapist; B: before treatment; A: after treatment; %: frequency of occurrence; NSBG: no significant bacterial growth; NOHE: National Orthopedic Hospital Enugu; UNTH: University of Nigeria Teaching Hospital; ESUTH: Enugu State University Teaching Hospital; CFU: colony forming unit.

**Table 7:** Carriage of microbial isolates by physiotherapists at UNTH, NOH, and ESUTH before and after hand wash (*N* = 19).

Isolates	UNTH (9 PTs)		NOHE (8 PTs)		ESUTH (2 PTs)	
	B	A	B	A	B	A
(i) *S. aureus*	44.44%	11.11%	37.50%	25.00%	50.00%	50.00%
(ii) *Coagulase* +ve* Staphylococcus*	33.33%	22.22%	12.50%	0.00%	50.00%	50.00%
(iii) *E. coli*	11.11%	0.00%	0.00%	0.00%	0.00%	50.00%
(iv) *Bacillus *spp.	11.11%	11.11%	0.00%	12.50%	0.00%	0.00%
(v) *Pseudomonas *spp.	0.00%	11.11%	0.00%	12.50%	0.00%	0.00%

*S. aureus*: *Staphylococcus aureus*; *coagulase *+ve: coagulase-positive; *E. coli*: *Escherichia coli*; spp.: species; PT: physiotherapist; B: before treatment; A: after treatment; %: frequency of occurrence; NSBG: no significant bacterial growth; NOHE: National Orthopedic Hospital Enugu; UNTH: University of Nigeria Teaching Hospital; ESUTH: Enugu State University Teaching Hospital.

**Table 8:** Susceptibility of bacterial isolates from hand contact swabs to common antibiotics.

Antibiotics	Concentration	Bacteria				
		*Staph. aureus *	*E. coli*	*Coagulase *+ve* Staphylococcus*	*Bacillus *spp.	*Pseudomonas *spp.
Streptomycin	30 mcg	66.7	66.7	40.5	100^*∗∗*^	33.3
Gentamicin	10 mcg	33.3	Rs	49.7	Rs	Rs
Ciprofloxacin	10 mcg	100.0^*∗∗*^	33.3	77.3	33.3	100^*∗∗*^
Lincomycin	30 mcg	33.3	Rs	54.1	Rs	34.7
Norfloxacin	30 mcg	Rs	Rs	41.7	Rs	Ns

Rifampicin	30 mcg	Rs	Rs	34.9	33.1	Ns
Chloramphenicol	30 mcg	Rs	Rs	37.3	Rs	33.3
Flucloxacillin	30 mcg	Rs	Rs	43.1	Rs	Rs
Erythromycin	30 mcg	Rs	Rs	47.7	100^*∗∗*^	55.7
Ampicillin-cloxacillin	10 mcg	33.3	Rs	40.3	33.3	Rs
Amoxicillin-clavulanic acid	10 mcg	Rs	Rs	43.7	Rs	Rs

Ofloxacin	10 mcg	33.3	Rs	75.7	33.3	100^*∗∗*^
Pefloxacin	30 mcg	Rs	Rs	82.1	Rs	100^*∗∗*^
Ampicillin	30 mcg	Rs	Rs	63.1	33.3	Rs
Cephalexin-nalidixic acid	30 mcg	Rs	Rs	72.5	Rs	33.3
Trimethoprim	30 mcg	Rs	Rs	80/2	Rs	Rs

mcg: microgram. ^*∗∗*^Figures represent percentage of isolates susceptible to drug; Rs: 100% of isolates resistant; 100^*∗∗*^: 100% of isolates susceptible to drug.
